# Smith's Duty to His Neighbour

**Published:** 1920-07-24

**Authors:** 


### THE PUBLIC WELL-BEING.

### Smith's Duty to his Neighbour.

### Presidential Address at Birmingham Health Congress.

The Congress of the Royal Sanitary Institute, which is in session this week at Birmingham, is providing a mine of valuable information on a very wide field of medicine, welfare, and allied subjects. We shall return again to the various matters discussed, but this week we must confine ourselves to the Presidential address by Viscount Astor, who dealt in terms of broad common sense with the atmosphere of public thought necessary for the healthy growth of new standards and new methods of public hygiene. He traced briefly our great advance in public sanitation, asked why, in that case, we are still so far from a healthy nation, and epitomised the development of his argument for progress upon the recognition of "Smith's responsibility to his neighbour if he has a cold." It is a theme well worth a Presidential address at an important health congress.

Why (asked Viscount Astor) do nearly half our deaths occur under fifty years of age? Why are only one in sixteen of our deaths due to old age? Why do deadly diseases, such as influenza, strike down 100,000 victims in a few months, while medical science seems to stand by almost powerless? One answer may be found in the immense crowding together of individuals which is part of modern civilisation. The isolation of the individual is almost a thing of the past. We may struggle to get more breathing-space for our houses, more air and sunshine in our windows, to bring the country nearer to the town and the factory more into the country; but the remorseless growth of population has us in its grip, and the complexity of civilisation interlocks even the peoples of distant countries. The daily, hourly contact of individuals presents comparatively new problems affecting modern health for which as a nation we have to find the solution. Nor is this the only new factor with which we have to deal. There is, indeed, a new world growing up around us—a world with new conventions, of changed social conditions, peopled by men and women of new outlook and with new ideas. Workers are getting higher wages and much more leisure. How they spend both their money and their time will have a bearing on national welfare. Greater freedom implies greater responsibility upon the individual, and the nation's need at the moment is for a development of the social conscience in the individual. We must look less to the community, to external environment, for the causes of national ill-health, and concentrate our efforts upon creating in the individual the same sense of personal health and responsibility as now exists in the community for communal services. "What we have now to recognise is *Smith's responsibility to his neighbour if he has a cold.*"

#### The Natural Aids.

The Ministry of Health can provide the means, but never the will, to health. It is the business of the Government to organise and to provide the instruments of good health; it is for the individual to utilise them. Everyone has more or less direct responsibilities in matters of health. He has to keep himself well; that is a primary duty of citizenship. He is responsible for preserving, as far as he can, the health of his family, his employees, and his co-workers. He must take his share in securing the welfare of the community in which he lives.

Among the greatest of "natural" aids to health are fresh air, sunlight, sleep, and cleanliness; yet how unnaturally are they neglected! Walk along a street (said the President) and observe the closed windows shutting out the air, the blinds barring out the sunshine, and in the evening weary little children losing precious hours of the sleep they need. In these matters alone how vast a field there is for the cultivation of knowledge, of a sense of individual responsibility and individual will. Nature, indeed, proposes, but man disposes with all his might and main. This is also true of the means provided by the State to meet the conditions of an artificial civilisation. Nature provides fresh air, the State requires windows, but only the individual can open them. The house may be small, even, alas! have too many occupants, yet its air can be fresher and purer than that of a half-empty mansion, and very often it is. Even in such a simple matter as fresh air, science has made quite recent progress, and is teaching us that movement, freshness, and coolness of air matters more than cubic capacity. The factory owner is required to provide such things as ventilators and safeguards to machinery, but their effectiveness depends upon the use that is made of them. The State can never compulsorily enforce maximum requirements; it can but insist upon a legal minimum, looking to the public spirit and intelligence of employers and employed to advance beyond the minimum. But if the minimum requirements are neglected or misused it is hopeless to expect any improvement upon them.

Maternity and child welfare centres are springing up in increasing number, but this number could be doubled or trebled if a strong public opinion desired it. The fact is that there are still a good many centres which are not used to the limit of their capacity. The will to health developed in the individual and expressed in the community is what is needed, together with a sense of responsibility towards the community in which he lives. The State has already granted to local authorities power to create maternity and child welfare centres, tuberculosis dispensaries, venereal disease centres, and other special instruments for preventive and curative medicine. But we shall never see real progress in public health until existing centres are fully utilised and until the individual sets himself to secure for his own locality and for the whole

**THE PUBLIC WELL-BEING**—(*continued*).

community the advantages which the State offers and which experience shows to be of such incalculable value.

The responsibility of the individual in the maintenance of his own health is really the keystone of the health structure which is slowly being created. The condition of our progress is an intelligent public opinion, a ready co-operation on the part of the individual. The business of the Health Ministry is to organise rather than to provide, and now to bring to the periphery the co-ordination that has been achieved at the centre.

Character is greater than the machine, and it is upon the national character that the future welfare of the country depends.

